# Low sun exposure increases multiple sclerosis risk both directly and indirectly

**DOI:** 10.1007/s00415-019-09677-3

**Published:** 2019-12-17

**Authors:** Anna Karin Hedström, Tomas Olsson, Ingrid Kockum, Jan Hillert, Lars Alfredsson

**Affiliations:** 1 Department of Clinical Neuroscience, Karolinska Institutet, 17177 Stockholm, Sweden; 2 Institute of Environmental Medicine, Karolinska Institutet, Stockholm, Sweden; 3 Centre for Occupational and Environmental Medicine, Stockholm County Council, Stockholm, Sweden

**Keywords:** Multiple sclerosis, Sun exposure, Vitamin D, Human leukocyte antigen, Interaction

## Abstract

**Objective:**

We aimed to study (1) to what extent the influence of low sun exposure on multiple sclerosis (MS) risk is mediated by low vitamin D levels; (2) whether low sun exposure or vitamin D deficiency act synergistically with HLA-DRB1*15:01 and absence of HLA-A*02:01.

**Methods:**

We used two population-based case–control studies (7069 cases, 6632 matched controls). Subjects with different HLA alleles, sun exposure habits and vitamin D status were compared regarding MS risk, by calculating odds ratios (OR) with 95% confidence intervals (CI) employing logistic regression. Mediation analysis was used to identify the potential mediation effect of vitamin D on the relationship between low sun exposure and MS risk.

**Results:**

Low sun exposure increased MS risk directly as well as indirectly, by affecting vitamin D status. The direct effect, expressed as OR, was 1.26 (95% CI 1.04–1.45) and the indirect effect, mediated by vitamin D deficiency, was 1.10 (95% CI 1.02–1.23). Of the total effect, nearly 30% was mediated by vitamin D deficiency. There was a significant interaction between low sun exposure and vitamin D deficiency (attributable proportion due to interaction 0.3, 95% CI 0.04–0.5) accounting for about 12% of the total effect. Further, both factors interacted with HLA-DRB1*15:01 to increase MS risk.

**Interpretation:**

Our findings indicate that low sun exposure acts both directly on MS risk as well as indirectly, by leading to low vitamin D levels. The protective effect of sun exposure thus seems to involve both vitamin D and non-vitamin D pathways, which is of relevance for prevention, in particular for those with a genetic susceptibility to MS.

**Electronic supplementary material:**

The online version of this article (10.1007/s00415-019-09677-3) contains supplementary material, which is available to authorized users.

## Introduction

MS is a chronic immune-mediated disease of the central nervous system with underlying genetic and environmental factors. The geographic variations in MS occurrence [1], and the changes in MS risk that occur with migration [2], have at least partly been attributed to differences in sun exposure levels [1, 2]. Several reviews have described consistent evidence of an association between both low sun exposure and vitamin D deficiency and increased MS risk [3, 4]. Exposure to sun leads to the release of a number of secondary mediators capable of suppressing cell-mediated immunity, and sun exposure also modulates immune responses by stimulating endogenous production of vitamin D [3, 4].

Interactions have been demonstrated between several lifestyle and environmental risk factors for MS and presence of the strongest genetic risk factor, the HLA-DRB1*15:01 allele [5-8]. A synergistic effect between vitamin D and DRB1*15:01 has been suggested [9], whereas we found no such interaction in a previous study comprising a relatively small case–control sample [10].

In the present study, based on two Swedish population-based case–control studies, we aimed to investigate to what extent the influence of low sun exposure on MS risk is mediated by vitamin D. We also aimed to study whether low sun exposure or vitamin D deficiency act synergistically with HLA-DRB1*15:01and absence of HLA-A*02:01 to increase MS risk.

## Methods

### Study design and study participants

Two population-based case–control studies on genetic and environmental factors for MS were used. One study was Genes and Environment in Multiple Sclerosis (GEMS) in which prevalent cases fulfilling the McDonald criteria [11] were identified from the Swedish National MS-registry [12]. One control per case was randomly selected from the national population register, matched for age, gender, and residential area at the time of disease onset. The study participants were recruited between November 2009 and November 2011.

The second study was Epidemiological Investigation of Multiple Sclerosis (EIMS) with a study group comprising the Swedish population aged 16–70 years. Incident cases of MS were recruited via neurology units in Swedish hospitals, including all university hospitals. Cases were diagnosed by a neurologist according to the McDonald criteria [11]. For each case, two controls were randomly selected from the national population register, matched by age in 5-year intervals, gender, and residential area. The study period was April 2005 to June 2015.

Patients in the Swedish MS registry who had previously been included in EIMS, were not included in GEMS. Randomly selected controls included in EIMS, were not used as controls in GEMS. Thus, there was no overlap of participants between the studies. The studies were approved by the Regional Ethical Review Board at Karolinska Institutet. All participants gave their informed consent to participate.

### Data collection

In both studies, information regarding environmental exposures and lifestyle factors was collected using a standardised questionnaire. The response rate was 82% for cases and 66% for controls in GEMS, and 93% for cases and 73% for controls in EIMS. Those who could not specify their sun exposure habits were excluded. All participants who filled out the questionnaire were asked to provide blood samples. Those who did not donate blood were excluded in the present report. The number of study subjects in each study is presented in supplementary Table 1.

### Genotyping and measurement of vitamin D

For all participants in both studies who donated blood, HLA-DRB1 and HLA-A alleles were determined at four-digit resolution. Genotyping was performed on the MS replication chip [13] which is based on an Illumina exome chip to which approximately 90,000 custom markers were added with extra high density in the HLA region and HLA alleles were then imputed with HLA*IMP:02 [14].

For EIMS participants recruited between 2005 and 2009 (*N* = 2464), vitamin D status was measured as levels of 25-hydroxy-vitamin D using a chemiluminescent immunoassay from Diasorin (Diasorin AB, Sundbyberg, Sweden) and a LIAISON® instrument provided by Diasorin AB with equimolar measurement of both 25-hydroxy-vitamin D_2_ and D_3_. The vitamin D analysis of the blood samples was performed in a single batch.

### Definition of sun exposure habits and vitamin D deficiency

In GEMS, information on outdoor activity during summer and winter in different age periods (0–9, 10–19, 20–29, 30–39, 40–49 and > 50 years) was used as a measure of sun exposure. The frequency of outdoor activity was reported on a four-point scale including ‘a couple of hours per day’, ‘a couple of hours per week’, ‘a couple of hours per month’, and ‘more seldom’. We constructed an index for sun exposure habits during the 10-year period prior to disease onset by adding the numbers from the four-point scales together, and thus acquired a value between 2 (the lowest exposure) and 8 (the highest exposure). Based on the 25th percentile among controls (six), sun exposure during the 10-year period prior to disease onset was dichotomized into high or low exposure.

We also dichotomized sun exposure during summer and winter, respectively, into high or low exposure defining high exposure as sun exposure 'a couple of hours per week' or 'a couple of hours per day' whereas less frequent sun exposure was defined as low sun exposure.

In EIMS, participants answered three questions regarding ultraviolet radiation (UVR) exposure during the 5-year period prior to inclusion in the study [11] (supplementary Table 2). Each answer alternative was reported on a four-point scale and we constructed an index by adding the numbers together and thus acquired a value between 3 (the lowest exposure) and 12 (the highest exposure). Sun exposure was dichotomized based on the 25th percentile among controls (five). Vitamin D deficiency was defined as a value less than 50 nmol/l.

### Statistical analysis

The studies were analyzed separately. Subjects with different sun exposure habits, vitamin D status and DRB1*15:01 status were compared with regard to MS risk by calculating OR with 95% CI using unconditional logistic regression models, with adjustment for the matching variables [15]. Trend tests for dose response relationships regarding degree of sun exposure/vitamin D levels and risk of MS were performed using continuous variables in a logistic regression model (index value between 2 and 8 in GEMS, and index value 3–12 in EIMS).

We categorized subjects based on amount of sun exposure (low/high) and vitamin D levels (low/high) and studied the influence of each factor in the absence of the other factor. When studying both factors simultaneously, causal mediation analysis was used to asses to what extent the relationship between low sun exposure and MS risk was mediated by vitamin D deficiency. The method allowed for exposure-mediator interaction. The causal effects were estimated on the OR scale and the CI were calculated using the delta method [16]. Further, a potential interaction on the additive scale was calculated between low sun exposure and vitamin D deficiency, using the attributable proportion due to interaction (AP) together with a 95% confidence interval.

The analyses were adjusted for age, gender, residential area, ancestry, smoking, adolescent BMI, infectious mononucleosis history, and the following MS-associated HLA alleles [17]; DRB1*03:01, DRB1*13:03, DRB1*08:01, B*44:02, B*38:01, B*55:01, DQA1*01:01, DQB1*03:02, DQB1*03:01, and homozygote correction for DRB1*15:01, DRB1*03:01 and A*02:01. When appropriate the analyses were also adjusted for DRB1*15:01, A*02:01 and sampling month.

Ancestry was dichotomized into Nordic versus non-Nordic origin. A participant who was born in any of the Nordic countries, whose parents had not immigrated from outside the Nordic countries, was classified as Nordic. Smoking was categorized into current, past or never smokers. Adolescent body mass index was calculated by dividing self-reported weight in kilograms by self-reported height in meters squared and dichotomized into more or less than 25 kg/m^2^. A history of infectious mononucleosis was dichotomized into yes or no.

Adjustments were also made for a number of potential confounding variables that only had minor influence on the results. These factors were not kept in the final analyses and included skin reactivity, intake of vitamin supplements, intake of fatty fish, educational level, passive smoking, and alcohol consumption. Skin reactivity was categorized into the following groups based on how the subjects’ skin react the first time they sunbathe in the summer without suntan lotion; ‘I always go red, never suntanned’, ‘I always go red, sometimes suntanned’, 'I sometimes go red, always suntanned’, ‘I never go red, get easily suntanned’, and ‘Always suntanned'. Intake of vitamin supplements was dichotomized into regular intake of multivitamins or vitamin D supplements or not. Intake of fatty fish was categorized into ‘never’, ‘1–3 times/months’, and ‘every week or daily’. Educational level was categorized into no post-secondary education, post-secondary education without a university degree, or university degree. Passive smoking was dichotomized into ever or never exposed. Using the same cutoffs as those used by Statistics Sweden, alcohol consumption was categorized into the following groups based on the amount of alcohol intake per week at the index year: no consumption, low consumption (< 50 g/week for women and < 100 g/week for men), moderate consumption (50–112 g/week for women and 100–168 g/week for men), or high consumption ((> 112 g/week for women and > 168 g/week for men). All analyses were conducted using Statistical Analysis System (SAS) version 9.4.

## Results

Our findings are based on 7069 cases and 6632 matched controls from two population-based case–control studies. The response rate was 82% for cases and 66% for controls in GEMS, and 93% for cases and 73% for controls in EIMS. The number of study subjects in each study is presented in supplementary Table 1. In both studies, the median age at MS onset was 33 years. In GEMS, the median duration from the disease onset to inclusion in the study was 17 years. Almost all cases in the EIMS study were recruited within 1 year after the diagnosis and the questionnaires were completed after a median of 3.0 years following the onset of the disease. Characteristics of cases and controls, by low versus high sun exposure at the index year, are presented in supplementary Table 3.

### Sun exposure, vitamin D levels, and occurrence of MS

In GEMS, low sun exposure both during summer and winter was associated with increased risk of MS (OR 1.3, 95% CI 1.0–1.8 and OR 1.2, 95% CI 1.0–1.5) (Table 1). In EIMS, low sun exposure during the 5-year period prior to study inclusion was associated with a 40% increased risk of MS (OR 1.4, 95% CI 1.1–1.7). In both studies, there was a trend showing increasing occurrence of MS with decreasing sun exposure (*p* values for trend < 0.0001).

**Table 1:** OR with 95% CI of MS for subjects with low sun exposure, compared to those with high sun exposure

Sun exposure (study)		Total		HLA data available		
		ca/co^a^	OR (95% CI)^b^	ca/co^a^	OR (95% CI)^b^	OR (95% CI)^c^
Sun exposure during the 10-year period prior to disease onset (GEMS)						
Overall	High Low	5478/4923 413/287	1.0 (reference) 1.3 (1.1–1.5)	4673/3539 339/210	1.0 (reference) 1.2 (1.0–1.5)	1.0 (reference) 1.3 (1.1–1.5)
Summer	High Low	5739/5122 152/88	1.0 (reference) 1.4 (1.2–1.9)	4889/3680 123/69	1.0 (reference) 1.3 (1.0–1.8)	1.0 (reference) 1.3 (1.0–1.8)
Winter	High Low	5449/4890 442/320	1.0 (reference) 1.2 (1.1–1.4)	4646/3514 336/235	1.0 (reference) 1.2 (1.0–1.4)	1.0 (reference) 1.2 (1.0–1.5)
Sun exposure during the 5-year period prior to study inclusion (EIMS)						
Overall	High Low	1749/4075 1058/1869	1.0 (reference) 1.3 (1.2–1.5)	1303/2006 754/877	1.0 (reference) 1.3 (1.2–1.5)	1.0 (reference) 1.4 (1.2–1.6)

^a^Number of exposed cases and controls

^b^Adjusted for gender, age, residential area, and ancestry

^c^adjusted for gender, age, residential area, ancestry, smoking, adolescent BMI, past IM, DRB1*15:01, DRB1*03:01, DRB1*13:03, DRB1*08:01, A*02:01, B*44:02, B*38:01, B*55:01, DQA1*01:01, DQB1*03:02, DQB1*03:01, and homozygote correction for DRB1*15:01, DRB1*03:01 and A*02:01

In EIMS, there was a trend showing increased MS risk with decreasing vitamin D levels (*p* value for trend 0.0001). When both sun exposure and vitamin D levels were run in the same logistic regression model as continuous variables, both trends remained significant. The results also remained similar when the analyses were restricted to include only subjects of Nordic ancestry.

The trend showing increased MS risk with decreasing vitamin D levels were restricted to subjects with vitamin D levels below 50 nmol/l. When the threshold of 50 nmol/l had been reached, vitamin D levels had no further significant impact on disease risk (supplementary Table 4).

### Causal mediation analysis in EIMS

The mediation analysis was based on EIMS and restricted to subjects with data on vitamin D levels. The total effect of low sun exposure on MS risk, expressed as OR, was 1.38 (95% CI 1.09–1.64). The direct effect was 1.26 (95% CI 1.04–1.45) and the indirect effect, mediated by vitamin D deficiency, was 1.10 (95% CI 1.02–1.23). Of the total effect, nearly 30% was mediated by vitamin D deficiency.

### Interaction between low sun exposure and vitamin D deficiency in EIMS

Compared with subjects with high sun exposure without vitamin D deficiency, there was a synergistic effect between low sun exposure and vitamin D deficiency among double-exposed subjects (AP 0.2, 95% CI 0.04–0.5) (Table 2). Interaction between low sun exposure and vitamin D deficiency accounted for approximately 12% of the total effect.

**Table 2:** OR with 95% CI of developing MS among subjects with different combinations of sun exposure habits during the 5-year period prior to study inclusion and vitamin D status

Sun exposure	Vitamin D deficiency	ca/co^a^	OR (95% CI)^b^	OR (95% CI)^c^	AP (95% CI)
High	No	539/695	1.0 (reference)	1.0 (reference)	
High	Yes	210/235	1.1 (0.9–1.4)	1.2 (1.0–1.6)	
Low	No	174/220	1.1 (0.8–1.3)	1.1 (0.8–1.4)	
Low	Yes	216/175	1.6 (1.3–2.0)	1.7 (1.3–2.1)	0.2 (0.04–0.5)

^a^Number of exposed cases and controls

^b^Adjusted for age, gender, residential area, and ancestry

^c^Adjusted for age, gender, residential area, ancestry, smoking, adolescent BMI, a history of IM, DRB1*03:01, DRB1*13:03, DRB1*08:01, A*02:01, B*44:02, B*38:01, B*55:01, DQA1*01:01, DQB1*03:02, DQB1*03:01, and homozygote correction for DRB1*15:01, DRB1*03:01 and A*02:01; *AP* attributable proportion due to interaction with 95% CI. The analysis is based on EIMS

### Interaction between low sun exposure, vitamin D deficiency, HLA-DRB1*15:01 and absence of HLA-A*02:01

In both studies, DRB1*15:01 interacted with low sun exposure with regard to MS risk (AP 0.3, 95% CI 0.1–0.6 in GEMS, and AP 0.2, 95% CI 0.1–0.4 in EIMS) (Table 3–4). The interaction remained significant when sun exposure during summer and winter was analyzed separately (Table 4). In EIMS, an interaction was also observed between DRB1*15:01 and vitamin D deficiency at study inclusion (AP 0.2, 95% CI 0.01–0.4) (Table 5). There was no interaction between low sun exposure or vitamin D deficiency and absence of A*02:01 (data not shown).

**Table 3:** OR with 95% CI of MS among subjects with different combinations of DRB1*15:01 status and sun exposure habits during the 10-year period prior to disease onset

DRB1*15:01 status	Overall sun exposure	ca/co^a^	OR (95% CI)^b^	OR (95% CI)^c^	AP (95% CI)
Negative	High	1947/2523	1.0 (reference)	1.0 (reference)	
Negative	Low	135/157	1.1 (0.9–1.4)	1.1 (0.9–1.4)	
Positive	High	2726/1016	3.5 (3.2–3.9)	3.7 (3.3–4.1)	
Positive	Low	204/53	5.1 (3.7–6.9)	5.9 (4.3–8.1)	0.3 (0.14–0.6)

DRB1*15:01 status	Summer sun exposure	ca/co^a^	OR (95% CI)^b^	OR (95% CI)^c^	AP (95% CI)
Negative	High	2034/2629	1.0 (reference)	1.0 (reference)	
Negative	Low	48/51	1.2 (0.8–1.8)	1.2 (0.8–1.7)	
Positive	High	2855/1051	3.6 (3.3–3.9)	3.8 (3.4–4.2)	
Positive	Low	75/18	5.4 (3.2–9.0)	6.0 (3.5–10.1)	0.3 (0.01–0.7)

DRB1*15:01 status	Winter sun exposure	ca/co^a^	OR (95% CI)^b^	OR (95% CI)^c^	AP (95% CI)
Negative	High	1929/2508	1.0 (reference)	1.0 (reference)	
Negative	Low	153/172	1.1 (0.9–1.4)	1.1 (0.9–1.4)	
Positive	High	2717/1006	3.6 (3.3–3.9)	3.7 (3.4–4.2)	
Positive	Low	213/63	4.5 (3.4–6.0)	5.2 (3.8–7.0)	0.3 (0.03–0.5)

^a^Number of exposed cases and controls

^b^Adjusted for age, gender, residential area, and ancestry

^c^Adjusted for age, gender, residential area, ancestry, smoking, adolescent BMI, a history of IM, DRB1*03:01, DRB1*13:03, DRB1*08:01, A*02:01, B*44:02, B*38:01, B*55:01, DQA1*01:01, DQB1*03:02, DQB1*03:01, and homozygote correction for DRB1*15:01, DRB1*03:01 and A*02:01; *AP* attributable proportion due to interaction with 95% CI. The analysis is based on GEMS

**Table 4:** OR with 95% CI of developing MS among subjects with different combinations of DRB1*15:01 and sun exposure habits during the 5 years prior to study inclusion

DRB1*15:01	Sun exposure	ca/co^a^	OR (95% CI)^b^	OR (95% CI)^c^	AP (95% CI)
Negative	High	577/1434	1.0 (reference)	1.0 (reference)	
Negative	Low	358/649	1.4 (1.2–1.6)	1.4 (1.2–1.6)	
Positive	High	726/572	3.2 (2.7–3.8)	3.7 (3.1–4.5)	
Positive	Low	396/228	4.4 (3.6–5.4)	5.3 (4.2–6.6)	0.2 (0.1–0.4)

^a^Number of exposed cases and controls

^a^Adjusted for age, gender, residential area, and ancestry

^c^Adjusted for age, gender, residential area, ancestry, smoking, adolescent BMI, a history of IM, DRB1*03:01, DRB1*13:03, DRB1*08:01, A*02:01, B*44:02, B*38:01, B*55:01, DQA1*01:01, DQB1*03:02, DQB1*03:01, and homozygote correction for DRB1*15:01, DRB1*03:01 and A*02:01; *AP* attributable proportion due to interaction with 95% CI. The analysis is based on EIMS

**Table 5:** OR with 95% CI of developing MS among subjects with different combinations of DRB1*15:01 and vitamin D deficiency

DRB1*15:01	Vitamin D deficiency	ca/co^a^	OR (95% CI)^b^	OR (95% CI)^c^	AP (95% CI)
Negative	No	307/660	1.0 (reference)	1.0 (reference)	
Negative	Yes	208/311	1.5 (1.2–1.9)	1.4 (1.1–1.8)	
Positive	No	406/255	3.4 (2.8–4.2)	4.4 (3.4–5.7)	
Positive	Yes	218/99	4.9 (3.7–6.4)	6.2 (4.5–8.6)	0.2 (0.01–0.4)

^a^Number of exposed cases and controls

^a^Adjusted for age, gender, residential area, and ancestry

^c^Adjusted for age, gender, residential area, ancestry, smoking, adolescent BMI, a history of IM, DRB1*03:01, DRB1*13:03, DRB1*08:01, A*02:01, B*44:02, B*38:01, B*55:01, DQA1*01:01, DQB1*03:02, DQB1*03:01, and homozygote correction for DRB1*15:01, DRB1*03:01 and A*02:01; *AP* attributable proportion due to interaction with 95% CI. The analysis is based on EIMS

## Discussion

Our findings indicate that low sun exposure may increase MS risk directly as well as indirectly, by affecting vitamin D status. Of the total effect, nearly 30% was mediated by vitamin D deficiency. There was a significant interaction between low sun exposure and vitamin D deficiency, accounting for about 12% of the total effect. Our findings indicate that low sun exposure and vitamin D deficiency are different risk factors that act synergistically to increase MS risk.

In Sweden, there is insufficient UVR to produce vitamin D during winter [18], yet we observed that sun exposure both during summer and winter was associated with MS risk, which further supports the view that the protective effects of sun exposure involve both vitamin D and non-vitamin D pathways [3, 4]. Sun exposure is the principal source of vitamin D and evidence indicates a protective role for higher vitamin D levels [19, 20]. However, a multi-ethnic study observed that sun exposure reduced the risk of MS regardless of race/ethnicity, whereas vitamin D deficiency was only associated with MS risk among whites [21]. Several studies have reported that sun exposure and vitamin D are independently associated with decreased MS risk [10, 22].

Apart from being essential for vitamin D production, sun exposure may result in suppression of cell mediated immunity, by the activation of regulatory T and B cells, and possibly by the range of cytokines and chemokines that are released following sun exposure [3, 23]. A suppressive effect of UVR through pathways independent of vitamin D have also been observed in experimental autoimmune enchephalomyelitis studies [24-26].

Both low sun exposure and vitamin D deficiency interacted with DRB1*15:01 to increase MS risk. Early studies have demonstrated that vitamin D influences HLA-DRB1 antigen expression and presentation [27]. There is a vitamin D response element (VDRE) region in the promoter region of HLA-DRB1 which is highly conserved on DRB1*15:01 haplotypes, whereas the VDRE present on other HLA-DRB1 haplotypes is not responsive to 1,25-dihydroxyvitamin D3 [9]. The interaction between low sun exposure/vitamin D deficiency and DRB1*15:01 indicates that these factors act on the immune system. The influence of several other lifestyle and environmental factors on MS risk is affected by the absence or presence of A*02:01 but this does not seem to be the case with sun exposure or vitamin D. The more restricted interaction between low sun exposure/vitamin D deficiency and HLA class II molecules suggests a different immune related pathway.

Both studies were designed as case–control studies in which information regarding environmental exposures was collected retrospectively. There is a risk of recall bias, especially when asking about exposures that are established risk factors for developing the disease. The risk of recall bias is greater in GEMS which uses prevalent MS cases. In EIMS, recall bias was minimized by predominantly including cases of MS who had received their diagnosis within the past year. However, our observation of an association between sun exposure and MS risk was similar in GEMS and EIMS, and also in agreement with numerous previous studies.

MS may result in sun avoidant behavior because of sensitivity to heat [28] which could lead to an overestimation of the inverse association between sun exposure during the 5-year period before study inclusion and MS risk in EIMS. However, the results remained similar when only subjects with disease onset during the past 2 years were included. In GEMS, low sun exposure during the 10-year period prior to disease onset was considered to minimize the potential problem of reverse causation. Furthermore, all main results remained similar when we instead considered the influence of low sun exposure during the age period 10–19 years. However, few cases and controls reported low exposure during this period.

The Swedish MS registry, through which the GEMS cases were recruited, is part of the clinical documentation system and used in all Swedish neurology departments and has been estimated to include data on approximately 83% of the prevalent MS patients in Sweden [12]. The response rate with regard to participation in GEMS was 82% for cases and 66% for matched controls. The relatively low participation rate among controls could introduce selection bias. However, selection bias is probably modest since the prevalence of lifestyle factors, such as smoking and socioeconomic status, among the controls was consistent with that of the general population in similar ages [29]. Furthermore, there were no significant differences with respect to age, gender, or sun exposure habits between those who provided a blood sample and those who did not, indicating that selection bias did not take place in this step. We consider it unlikely that our findings would be affected by bias to a large extent, especially since such a bias would then depend both on HLA genotype and sun exposure habits.

In EIMS, a sun exposure index, ranging between 3 and 12, was used as a measure of sun exposure. In our previous study on sun exposure and MS risk, based on EIMS, we used a higher cut-off (the median among controls) when defining high and low sun exposure habits, and we observed no synergistic effect between low sun exposure and DRB1*15:01 with regard to MS risk [10]. In the present study, which has a larger number of cases and controls, low sun exposure was defined as a value below the 25th percentile among controls. The results from the former study based on EIMS are thus not comparable with the results based on EIMS in the present study.

Vitamin D levels were measured after MS onset and these levels were assumed to reflect levels before disease onset. Therefore, we only show an association, without being able to confirm causality. However, our findings remained similar when we restricted the analysis to include only subjects with disease onset within the past year. The observed interaction between low sun exposure/vitamin D deficiency and DRB1*15:01 also alleviates the magnitude of some potential biases in the interpretation of the influence from these lifestyle factors on MS risk, since DRB1*15:01 is not likely to determine exposure habits. We thus consider it unlikely that our findings would be affected by bias to a large extent, especially since such a bias would then depend on HLA types.

In the present study, we had the opportunity to take a large number of potential confounding factors into account, including skin reactivity to sun exposure, vitamin supplements, intake of fatty fish, smoking, education and genetics. The factors considered only had minor influence on the results. However, we cannot rule out that other factors related to sun-exposure or the studied HLA genes partly could influence the magnitude or the estimated measure of interaction.

In conclusion, our findings indicate that low sun exposure may increase MS risk directly as well as indirectly, by affecting vitamin D status. We also found signs of interactions between both low sun exposure and vitamin D deficiency and DRB1*15:01 in relation to MS risk. The potential protective effect of sun exposure thus seems to involve both vitamin D and non-vitamin D pathways, which is of relevance for prevention, in particular for those with a genetic susceptibility to MS. Modest sun exposure habits could thus be recommended.

## Electronic supplementary material

Below is the link to the electronic supplementary material.

Supplementary file1 (DOC 50 kb)
