# Supplementary material for: Low sun exposure increases multiple sclerosis risk both directly and indirectly
Source: J Neurol. 2019 Dec 17;267(4):1045–52. doi: 10.1007/s00415-019-09677-3 (PMC7109160; doi:10.1007/s00415-019-09677-3)
Supplement: Supplementary file 1 — Supplementary file1 (DOC 50 kb) [file 415_2019_9677_MOESM1_ESM.doc]

Supplementary table 1. Number of cases and controls included in the study.

| Study | | Included in the study | Data on environmental exposures | HLA genotype available. Dataset for analysis. |
| --- | --- | --- | --- | --- |
| GEMS | Cases | 6156 | 5891 | 5012 |
|  | Controls | 5408 | 5210 | 3749 |
| EIMS | Cases | 2880 | 2807 | 2057 |
|  | Controls | 6122 | 5944 | 2883 |

Supplementary table 2. Questions regarding sun exposure habits in EIMS.

1. How often, during the last 5 years, have you used a sun-bed?

Never A few times a year Once a month Once a week

2. How often, during the last 5 years, have you visited a country that is sunnier than Sweden?

Never More seldom Once a year More than once a year

3. If the weather is sunny, how often do you usually sunbathe?

Never A few times a month A few times a week Daily

Supplementary table 3. Characteristics of cases and controls in GEMS and EIMS, by sun exposure habits at the index year.

| Study | GEMS | | | | | | EIMS | | | | | |
| --- | --- | --- | --- | --- | --- | --- | --- | --- | --- | --- | --- | --- |
|  | Sun exposure during the 10-year period prior to disease onset | | | | | | Sun exposure during the 5-year period prior to study inclusion | | | | | |
|  | Total | | Low | | High | | Total | | Low | | High | |
|  | Cases | Controls | Cases | Controls | Cases | Controls | Cases | Controls | Cases | Controls | Cases | Controls |
| Women, n (%) | 3642 (73) | 2883 (77) | 1056 (73) | 849 (77) | 2586 (73) | 2034 (77) | 1490 (72) | 2164 (75) | 483 (64) | 570 (65) | 1007 (77) | 1594 (79) |
| Men, n (%) | 1370 (27) | 866 (23) | 395 (27) | 250 (23) | 975 (27) | 616 (23) | 567 (28) | 719 (25) | 271 (36) | 307 (35) | 296 (23) | 412 (21) |
| Nordic, n (%) | 4323 (86) | 3251 (87) | 1243 (86) | 926 (84) | 3080 (86) | 2325 (88) | 1681 (82) | 2288 (79) | 621 (82) | 712 (81) | 1060 (81) | 1576 (79) |
| Smoking, n (%) | 2856 (57) | 1828 (49) | 868 (60) | 552 (50) | 1988 (56) | 1276 (48) | 1092 (53) | 1283 (45) | 406 (54) | 353 (40) | 686 (53) | 930 (46) |
| Body mass index >25 kg/m^2^, n (%) | 557 (11) | 326 (8.7) | 165 (11) | 97 (8.8) | 392 (11) | 229 (8.6) | 354 (17) | 305 (11) | 152 (20) | 111 (13) | 202 (16) | 194 (10) |
| Infectious mononucleosis, n (%) | 595 (12) | 251 (6.7) | 175 (12) | 76 (6.9) | 420 (12) | 175 (6.6) | 355 (17) | 290 (10) | 111 (15) | 77 (8.8) | 244 (19) | 213 (11) |
| Total, n | 2012 | 3749 | 1451 | 1099 | 3561 | 2650 | 2057 | 2883 | 753 | 876 | 1300 | 2005 |

Supplementary table 4. OR with 95% CI of developing MS among subjects with different vitamin D levels, compared to those with vitamin D levels of 75 or more.

| Vitamin D levels | ca/co | OR (95% CI) | OR (95% CI) |
| --- | --- | --- | --- |
| 75+ | 310/414 | 1.0 (reference) | 1.0 (reference) |
| 70-74 | 62/87 | 1.0 (0.7-1.4) | 1.0 (0.7-1.5) |
| 65-69 | 92/112 | 1.1 (0.8-1.5) | 1.0 (0.7-1.4) |
| 60-64 | 90/113 | 1.1 (0.8-1.5) | 1.0 (0.7-1.4) |
| 55-59 | 74/91 | 1.1 (0.8-1.6) | 1.1 (0.7-1.5) |
| 50-54 | 89/107 | 1.1 (0.8-1.6) | 1.1 (0.8-1.5) |
| 45-49 | 88/94 | 1.3 (0.9-1.8) | 1.3 (0.9-1.8) |
| 40-44 | 84/87 | 1.3 (1.0-1.9) | 1.4 (1.0-2.0) |
| 35-39 | 77/77 | 1.4 (1.0-2.0) | 1.4 (0.9-2.0) |
| 30-34 | 58/52 | 1.5 (1.0-2.3) | 1.4 (0.9-2.2) |
| <30 | 115/91 | 1.7 (1.3-2.4) | 1.8 (1.2-2.5) |
